# Cultivar-specific metabolomic and morpho-physiological responses reveal distinct salt tolerance mechanisms in rice (Oryza sativa L.)

**DOI:** 10.1186/s12870-025-08086-1

**Published:** 2026-02-02

**Authors:** Nagy S. Radwan, Sobhi F. Lamlom, Abdul-Hamid Emwas, Mariusz Jaremko, Nader R. Abdelsalam

**Affiliations:** 1 Agricultural Botany Department, Faculty of Agriculture, Saba Basha, Alexandria University, Alexandria, 21531 Egypt; 2 Department of Plant Production, Faculty of Agriculture Saba Basha, Alexandria University, Alexandria, Egypt; 3 Core Lab of NMR, King Abdullah University of Science and Technology (KAUST), Thuwal, Makkah 23955-6900 Saudi Arabia; 4 Smart-Health Initiative (SHI) and Red Sea Research Center (RSRC), Division of Biological and Environmental Sciences and Engineering (BESE), King Abdullah University of Science and Technology (KAUST), Thuwal, Makkah 23955-6900 Saudi Arabia

**Keywords:** Rice, Salt stress, Metabolites, GC-MS, Cultivar tolerance, Osmotic adjustment

## Abstract

Salt stress significantly limits rice productivity worldwide, highlighting the need for a thorough understanding of cultivar-specific tolerance mechanisms. This study explored the morpho-physiological and metabolomicresponses of four rice cultivars to salinity stress, aiming to identify potential adaptation strategies and candidate tolerance markers. The four rice cultivars (Giza 177, Giza 178, Sakha 104, and Sakha 108) were subjected to 200 mM NaCl treatment for 14 days under controlled conditions. Morpho-physiological parameters measured included fresh weight, relative water content (RWC), proline content, and plant height. Comprehensive metabolomic profiling was performed using GC-MS analysis of leaf and root tissues, followed by multivariate statistical analyses, including principal component analysis (PCA) and variable importance in projection (VIP) scores. Salt stress significantly decreased fresh weight and RWC across all cultivars (*p* < 0.001) and led to substantial proline accumulation. Sakha 108 showed superior salt tolerance (78.6% tolerance index), maintaining higher biomass and water content under stress conditions. PCA distinguished control from salt-treated samples (PC1: 75.2% variance). Metabolomic analysis identified 114 metabolites in leaf tissues and 97 in roots, with 40 metabolites exhibiting VIP scores greater than 1. Cultivar-specific responses included enhanced amino acid metabolism in Giza 177 and activated lipid metabolism pathways in Sakha 108. Hierarchical clustering highlighted tissue-specific metabolic reprogramming, with only six metabolites displaying conserved expression patterns across all cultivars in leaves. The rice cultivars employed different metabolic strategies for salt tolerance, with Sakha 108 demonstrating integrated mechanisms that combine osmotic adjustment, metabolic flexibility, and water retention. These findings offer initial insights for breeding salt-tolerant rice varieties and understanding plant stress adaptation at the metabolomic level within this experimental system.

**Supplementary Information:**

The online version contains supplementary material available at 10.1186/s12870-025-08086-1.

## Introduction

Rice (*Oryza sativa* L.) is widely cultivated all over the globe because of its wider adaptability and popularity as a carbohydrate-rich diet [1]. Rice provides primary calories and is a good source of micronutrients and macronutrients, including proteins, lipids, carbohydrates, dietary fibers, minerals, and vitamins [2, 3]. Rice is cultivated on over 164 Mha of land in more than 100 countries annually [4]. The global demand for rice is expected to grow to 852 million tons by 2035 as the yield per unit area increases year by year [5]. To meet demand, rice production must rise by at least 1% annually [6]. Conversely, environmental stressors have been identified as the leading causes of declining agricultural output in quantity and quality.

Environmental stress responses in plants involve complex biochemical cascades where metabolic intermediates act as key modulators and phenotypic effectors. These bioactive compounds play dual roles: regulating morphophysiological adaptations and mediating genotype-phenotype relationships [7]. Metabolomic profiling allows detailed analysis of metabolic changes and helps clarify how plants respond to stress, making it a vital tool for studying plant-environment interactions [8].

Secondary metabolite biosynthesis is a key part of plant cellular processes, with these compounds playing essential roles in signal transduction, defense mechanisms, and maintaining physiological balance [9–11]. Current estimates suggest that plant species collectively produce over 200,000 distinct secondary metabolites, highlighting their biological importance [12, 13]. Primary metabolic constituents, such as carbohydrates, amino acids, nucleotides, and organic acids, work together with secondary metabolites like alkaloids, phenolic compounds, quinones, flavonoids, and terpenoids to support stress tolerance [13, 14].

Salt stress represents one of the most significant abiotic constraints affecting global agricultural productivity, with approximately 20% of cultivated land and 50% of irrigated areas worldwide experiencing varying degrees of salinity [15, 16]. Rice, as one of the world’s most important staple crops, faces increasing challenges from soil salinization due to climate change, sea-level rise, and intensive agricultural practices [17, 18]. Unlike drought stress, salt stress imposes both osmotic and ionic toxicity on plants, creating a burden that significantly impacts cellular metabolism, growth, and development [19].

The metabolic response of rice to salt stress involves complex biochemical adjustments across primary and secondary metabolic pathways. Under saline conditions, rice plants experience disrupted ion homeostasis, particularly excessive accumulation of Na⁺ and Cl⁻ ions, which interfere with essential physiological processes, including photosynthesis, protein synthesis, and enzyme activity [9]. To counteract these detrimental effects, rice plants activate sophisticated metabolic defense mechanisms, including the synthesis of compatible solutes such as proline, glycine betaine, and trehalose, which help maintain cellular osmotic balance and protect macromolecular structures [20]. The phenolic compounds, flavonoids, and other secondary metabolites play crucial roles in salt stress tolerance by functioning as antioxidants to mitigate oxidative damage caused by reactive oxygen species (ROS) accumulation [21]. Additionally, metabolic reprogramming under salt stress involves alterations in amino acid metabolism, carbohydrate partitioning, and lipid composition, which collectively contribute to the plant’s adaptive response [22]. Understanding these metabolic changes through comprehensive metabolomics approaches provides valuable insights into the molecular mechanisms of salt tolerance, offering potential targets for developing salt-resistant rice varieties.

This study explores how different rice genotypes exhibit unique metabolic signatures related to salt tolerance. By focusing on metabolic plasticity, we analyze how it supports adaptive responses to various salinity levels and stress durations. Using GC-MS, we profiled the metabolism of leaves and roots from four rice varieties, Giza 177, Giza 178, Sakha 104, and Sakha 108, under both normal and saline conditions, alongside measuring morphological and physiological traits. The results show notable differences among varieties in their metabolic responses to salt stress. These insights improve our understanding of the specific adaptive strategies rice employs in saline environments.

## Materials and methods

### Plant material and growth conditions

Four *Oryza sativa* L. cultivars were obtained from the Rice Research Department, Sakha Agricultural Research Station, Kafr El-Sheikh, Egypt (Table 1). The cultivars were selected based on their documented contrasting salt tolerance characteristics, as established in previous research, and their agricultural importance in the Middle East and North Africa region. Sakha 108 and Giza 178 were chosen as representatives of salt-tolerant genotypes, while Giza 177 was selected as a salt-sensitive control. Sakha 104, known primarily for its drought sensitivity, was included to investigate potential cross-stress tolerance mechanisms and metabolic pathway overlaps among different abiotic stresses.

**Table 1:** Genetic background and stress tolerance characteristics of rice cultivars used in this study

No.	Variety	Pedigree	Type	Stress Tolerance	Reference
1	Sakha104	GZ 4096-8-1/GZ 4100-9-1	Japonica	Drought-sensitive	[25]
2	Sakha108	Sakha 101/HR 5824-B-3-2-3//Sakha 101	Japonica	Salt-tolerant	[26, 27]
3	Giza 177	Not available	Indica/Japonica	Salt-sensitive	[26, 27]
4	Giza 178	Giza 175/Milyang 49	Indica/Japonica	Salt-tolerant	[28]

Seeds were surface-sterilized with a 3% (v/v) sodium hypochlorite solution for 10 min, followed by three rinses with sterile distilled water to remove residual sterilizing agent. Sterilized seeds were placed on moist filter paper (Whatman No. 1) in petri dishes and incubated at room temperature (25 ± 2 °C) in darkness for germination. Pre-germinated seeds showing uniform radicle emergence (≥ 2 mm) from each genotype were selected and sown in plastic trays (30 × 20 × 10 cm) containing a sterilized mixture of field soil and farmyard manure (3:1, v/v) [23, 24].The growth medium was prepared by autoclaving the soil-manure mixture at 121 °C for 15 min and allowing it to cool before use. Seedlings were subsequently transferred to a controlled growth chamber (Conviron CMP6010, Controlled Environments Ltd., Winnipeg, Canada) maintained at 70 ± 5% relative humidity and 24 ± 2 °C day/night temperature with a 16-hour photoperiod under photosynthetic photon flux density of 250–350 µmol m⁻² s⁻¹ provided by LED panels (Philips GreenPower LED Production Module).

### Experimental design and salt stress treatment

The experiment was set up using a factorial randomized complete block design (RCBD) with four biological replicates. Factor A included the four rice cultivars (Giza 177, Giza 178, Sakha 104, and Sakha 108), while Factor B consisted of two treatment conditions: control (distilled water) and salt stress (200 mM NaCl solution). The choice of 200 mM NaCl was based on an extensive literature review and preliminary testing to identify a salinity level that effectively distinguishes between tolerant and sensitive genotypes while remaining biologically relevant. This concentration, with an electrical conductivity of approximately 11.7 dS/m, represents moderate salinity stress commonly found in salt-affected agricultural soils worldwide.

Salt stress treatment was initiated at the two-leaf stage and continued for 14 consecutive days. Each experimental unit contained 20 seedlings, with treatments applied through irrigation with the respective solutions every 48 h at a rate of 50 mL per tray to maintain consistent soil moisture without waterlogging. Control plants received an equivalent volume of distilled water following the same irrigation schedule. Environmental conditions were continuously monitored throughout the experiment using data loggers (HOBO MX2301A, Onset Computer Corporation, Bourne, MA, USA) to maintain stable growth conditions. All tissue samples were harvested at Zeitgeber time 6 h (6 h after lights-on, corresponding to mid-morning) to minimize circadian effects on metabolite profiles. Sampling was completed within a 2-hour window under consistent light conditions (250–350 µmol m⁻² s⁻¹ PPFD) to ensure comparability across all treatments and biological replicates.

### Morpho-physiological and biochemical analyses

#### Plant height, root biomass measurements, and relative water content

Plant height (PH) was measured from the soil surface to the tip of the longest leaf using a standard ruler and expressed in centimeters.

Root fresh weight (RFW) was measured at harvest (14 days after salt stress initiation) by carefully removing plant roots from the growth medium, gently washing them with distilled water to remove adhering soil particles, blotting excess water with tissue paper, and immediately weighing on an analytical balance (± 0.001 g). For dry weight determination, root samples were oven-dried at 85 °C until constant weight was achieved to obtain root dry weight (RDW). Shoot fresh weight (SFW) was determined immediately after harvesting by severing the shoots at the crown, removing any extraneous material, and weighing them on the same analytical balance. These shoot samples were then oven-dried at 85 °C alongside the roots until a constant weight was achieved to obtain shoot dry weight.

Relative water content (RWC) was determined following the method of Barrs and Weatherley [29]. Fresh leaf discs (1 cm²) were weighed immediately after collection (FW), then floated in distilled water for four hours at room temperature to achieve full turgidity before reweighing (TW). Subsequently, discs were oven-dried at 85 °C until constant weight was achieved (DW). RWC was calculated using the formula:

$$\:RWC\:\left(\%\right)\:=\:\frac{(FW\:-\:DW)}{(TW\:-\:DW)}\:\times\:\:100$$

Where: FW = Fresh weight; TW = Turgid weight; DW = Dry weight.

#### Chlorophyll content

Total chlorophyll content was estimated using a portable chlorophyll meter (SPAD-502, Minolta Co., Osaka, Japan) and expressed as SPAD units. Measurements were taken from ten randomly selected fully expanded leaves per treatment at 14 days after stress initiation, following the protocol described by Uddling et al. [30].

#### Proline content determination

Proline content was quantified using a modified ninhydrin-based colorimetric method [31]. Fresh leaf tissue (100 mg) was homogenized in 3% (w/v) sulfosalicylic acid (5 µL mg⁻¹ fresh weight) and centrifuged at 12,000× *g* for 5 min at 4 °C. The supernatant (100 µL) was mixed with an equal volume of reaction mixture containing 3% sulfosalicylic acid, glacial acetic acid, and acidic ninhydrin (1:2:2, v/v/v). The mixture was incubated at 96 °C for 60 min, followed by toluene extraction (1 mL). After phase separation, the chromophore-containing toluene layer was collected, and absorbance was measured at 520 nm using a NanoDrop 2000 spectrophotometer (Thermo Scientific, Wilmington, DE, USA) [32]. Proline concentration was calculated using a standard curve and expressed as µmol g⁻¹ fresh weight using the formula [33]:

$$\begin{aligned}& \mathbf{Proline}\;(\boldsymbol{\upmu}\mathbf{mol\;g}^{-1}\;\mathrm{FW})\;\\&=\;\frac{[(\upmu\mathrm{g}\;\mathrm{proline}\;\mathrm{mL}^{-1}\;\times\;\mathrm{mL}\;\mathrm{toluene})\;/\;115.5]}{(\mathrm{g}\;\mathrm{FW}\;/\;5)} \end{aligned}$$

Where: 115.5 = molecular weight of proline; Values derived from standard curve.

### Metabolite extraction and GC-MS analysis

#### Metabolite extraction

Metabolite extraction was performed using a methanol-chloroform-based protocol optimized for the recovery of polar metabolites. Leaf and root tissues were immediately frozen in liquid nitrogen after harvest and stored at -80 °C until analysis. Frozen tissue samples (100 mg fresh weight) were ground to fine powder using a pre-chilled mortar and pestle with liquid nitrogen. The ground tissue was transferred to a 2 mL microcentrifuge tube and extracted with 1 mL of ice-cold extraction solvent consisting of methanol: chloroform: water (5:2:2, v/v/v) containing ribitol (20 µg/mL; Sigma-Aldrich, St. Louis, MO, USA) as internal standard for normalization of extraction efficiency and instrument response variations. Samples were vortexed vigorously for 30 s and incubated at 4 °C with continuous shaking (1,400 rpm) for 15 min. Following incubation, samples were centrifuged at 14,000× g for 10 min at 4 °C to pellet cellular debris. The supernatant was carefully transferred to a clean glass vial. For phase separation, 400 µL of ultrapure water (Milli-Q, Millipore, Bedford, MA, USA) was added to each sample, followed by vigorous vortexing for 10 s and centrifugation at 2,200× g for 5 min at 4 °C. The upper polar phase (methanol-water layer, approximately 600 µL) was transferred to a 1.5 mL glass vial with a polytetrafluoroethylene (PTFE)-lined cap.

#### Sample derivatization

The polar extracts were dried completely under a gentle stream of nitrogen gas at 37 °C using a sample concentrator. To ensure complete removal of residual water, samples were further dried under vacuum in a SpeedVac concentrator (Thermo Fisher Scientific, Waltham, MA, USA) for 30 min. Dried samples were derivatized using a two-step procedure. First, carbonyl groups were protected by methoximation: 40 µL of methoxyamine hydrochloride solution (20 mg/mL in anhydrous pyridine; Sigma-Aldrich) was added to each dried sample. Vials were capped, vortexed for 30 s, and incubated at 37 °C for 90 min with continuous shaking. Second, trimethylsilylation was performed by adding 70 µL of N-methyl-N-(trimethylsilyl) trifluoroacetamide (MSTFA) with 1% trimethylchlorosilane (TMCS; Sigma-Aldrich) to each sample. Vials were immediately capped, vortexed for 30 s, and incubated at 60 °C for 60 min. After cooling to room temperature, samples were briefly centrifuged (2,000× g, 1 min) and transferred to GC autosampler vials with glass inserts for immediate analysis.

#### GC-MS analysis

Gas chromatography-mass spectrometry analysis was conducted using an Agilent 7890B gas chromatograph coupled with a 5977 A mass selective detector (Agilent Technologies, Santa Clara, CA, USA). Chromatographic separation was achieved using a DB-5MS capillary column (30 m × 0.25 mm inner diameter × 0.25 μm film thickness; Agilent J&W Scientific, Folsom, CA, USA) with a 10 m Duraguard pre-column. One microliter of derivatized sample was injected in splitless mode with an injection port temperature of 250 °C. The splitless time was set to 1 min, after which the split ratio was adjusted to 20:1. Helium (99.9999% purity) was used as carrier gas at a constant flow rate of 1.2 mL/min. The oven temperature program was as follows: initial temperature of 70 °C held for 2 min, ramped at 5 °C/min to 310 °C, and held at 310 °C for 12 min, resulting in a total run time of 62 min per sample. Mass spectrometry was performed in electron ionization (EI) mode at 70 eV. The ion source temperature was maintained at 230 °C and the quadrupole temperature at 150 °C. The transfer line temperature was set at 280 °C. The mass spectrometer was operated in full scan mode with a mass range of 50–600 m/z and a scan rate of 2.7 scans per second. A solvent delay of 6 min was applied to protect the detector filament.

#### Quality control and system performance monitoring

Quality control samples were prepared by pooling equal volumes of all biological samples after derivatization. A QC sample was injected at the beginning of the sequence, after every 10 sample injections, and at the end of the analytical sequence to monitor instrument performance and metabolite stability. The correlation coefficients (R²) for quality control samples consistently exceeded 0.97, indicating high data reliability across all samples. Blank samples (containing only derivatization reagents) were injected at the beginning and end of each batch to assess background contamination. Additionally, a mixture of n-alkane standards (C10-C40; Sigma-Aldrich) was run at the beginning of each batch to calculate retention indices for metabolite identification. System suitability was confirmed by monitoring the retention time stability (± 0.05 min), peak area reproducibility (relative standard deviation < 15% for major peaks in QC samples), and signal-to-noise ratio (> 10:1 for major metabolite peaks).

#### Metabolite identification and data processing

Metabolite identification was achieved by comparing with the NIST 17 mass spectral library (National Institute of Standards and Technology, Gaithersburg, MD, USA) using Agilent MassHunter Qualitative Analysis software (Version B.08.00). Identification criteria included a minimum spectral match factor of 80% and a retention time deviation of less than 5% compared to authentic standards when available. Peak integration was performed using Agilent MassHunter Quantitative Analysis software (Version B.09.00), with manual verification of integration boundaries. Quality control measures involved the use of ribitol (Sigma-Aldrich) as an internal standard at a final concentration of 10 µg/mL to normalize extraction efficiency and instrument response variations.

Raw data preprocessing involved filtering compounds based on peak intensity thresholds (minimum of 1000 counts) and their presence in at least 80% of samples within each treatment group. Missing values were addressed using K-nearest neighbor (KNN) imputation for metabolites with less than 20% missing data across all samples. In contrast, metabolites exceeding this threshold were removed from the analysis. Data normalization was performed using log₂ transformation, followed by auto-scaling (mean-centering and unit-variance scaling) to account for differences in metabolite concentration ranges and ensure equal weight in multivariate analyses.

### Statistical analysis

Statistical analyses were performed using MetaboAnalyst 5.0 (https://www.metaboanalyst.ca/, accessed on March 20, 2025) and R software (Version 4.3.0, R Foundation for Statistical Computing, Vienna, Austria). Morpho-physiological data were analyzed using a two-way analysis of variance (ANOVA) with cultivar and treatment as fixed factors, and biological replicates were treated as random effects. Normality and homoscedasticity assumptions were verified using the Shapiro-Wilk and Levene’s tests, respectively. When significant differences were found, means were compared using Tukey’s honestly significant difference (HSD) test at α = 0.05. For metabolomic data, both univariate and multivariate statistical analyses were applied. Univariate analysis involved unpaired t-tests for pairwise comparisons between treatments within each cultivar, with multiple testing corrections using the Benjamini-Hochberg false discovery rate (FDR) procedure to reduce Type I errors. Significance was defined as FDR-adjusted *p* < 0.05. Effect sizes were measured using Cohen’s d for all significant metabolite differences, with 95% confidence intervals calculated via bootstrap resampling (*n* = 1000). Multivariate analyses included hierarchical cluster analysis (HCA), principal component analysis (PCA), and partial least squares discriminant analysis (PLS- DA). PCA was conducted on mean-centered and unit-variance-scale data to identify key sources of variation in metabolomic profiles. Orthogonal Partial Least Squares Discriminant Analysis (OPLS-DA) was employed as a supervised multivariate method to maximize separation between predefined groups (control vs. salt-treated) and identify discriminant metabolites. OPLS-DA models were constructed with one predictive component and one or more orthogonal components to separate treatment-related variation from within-class variation. Model quality was assessed using R²X (cumulative explained variation in metabolite data), R²Y (cumulative explained variation in class membership), and Q² (cumulative predictive ability based on seven-fold cross-validation) parameters. Model validity was rigorously tested using permutation testing (*n* = 200 permutations) to ensure that the observed separation was not due to chance. R² indicated a valid model, and Q² intercepts less than 0.3 and 0.05, respectively, in permutation plots. Additionally, a cross-validated ANOVA (CV-ANOVA) was performed to assess the statistical significance of the OPLS-DA model (*P* < 0.05, indicating a significant model). Variable importance in projection (VIP) scores were calculated from the OPLS-DA model to identify metabolites most responsible for group discrimination. Metabolites with VIP scores ≥ 1.0 were considered important contributors to group separation, and a VIP threshold of > 1.6 was used to identify the most discriminatory biomarkers. Hierarchical clustering was performed using Euclidean distance and Ward’s linkage method to reveal clustering patterns among samples and metabolites. Bootstrap validation (*n* = 1000) was used to assess the stability of multivariate models and ensure reliable interpretation. Post-hoc power analysis confirmed that all significant differences had statistical power greater than 0.8 to detect meaningful biological effects. Data are shown as means ± standard error of the mean (SEM) unless otherwise noted. All statistical tests used a significance threshold of *p* < 0.05. Experimental procedures complied with institutional guidelines and regulations for plant research.

## Results

### Morpho-physiological responses to salt stress

**Fig. 1:**
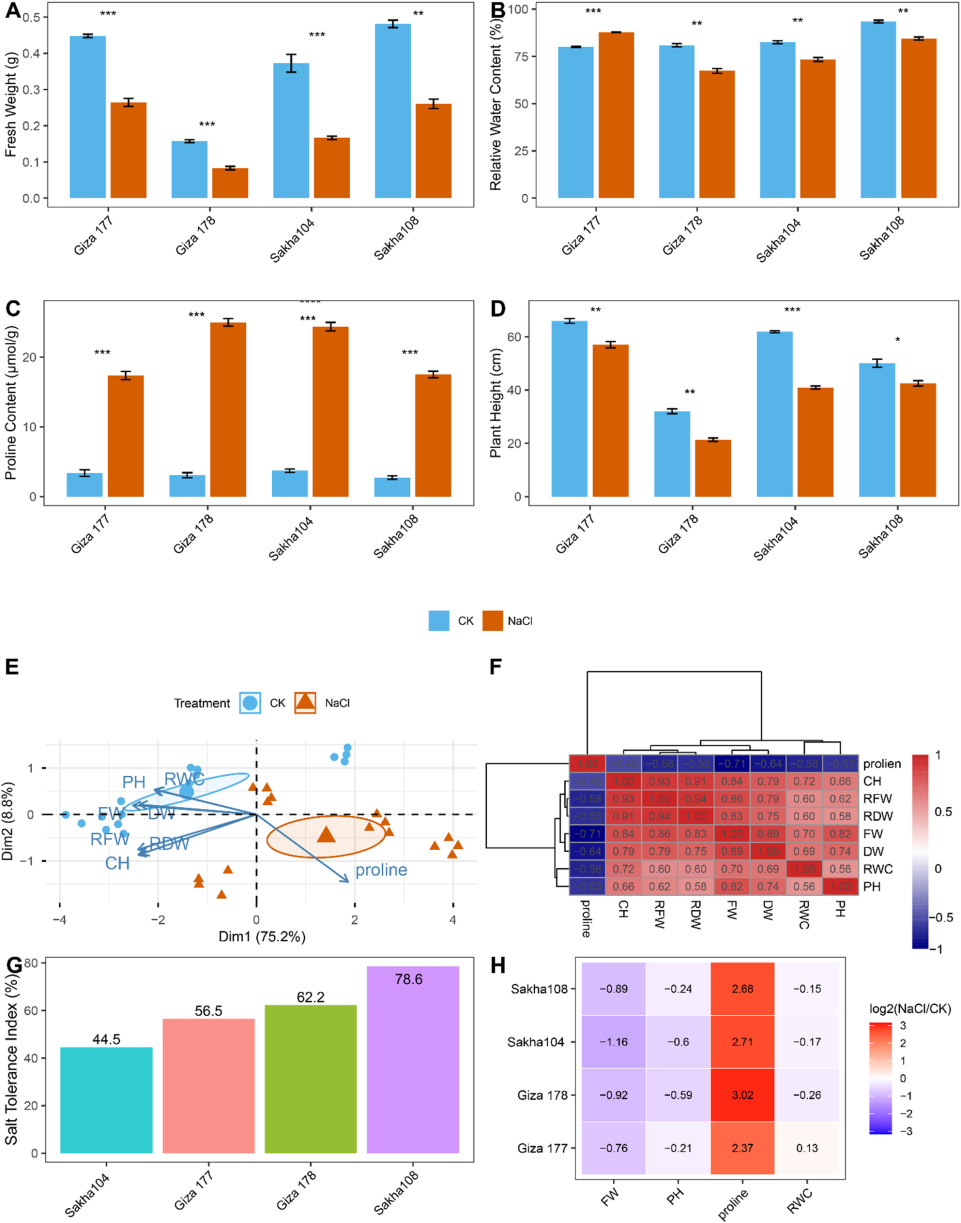
Differential responses of rice cultivars to salt stress treatments. **A** Fresh weight of rice seedlings under control (CK) and salt stress (NaCl) conditions. **B** Relative water content (RWC) of leaves in response to salt treatment. **C** Proline accumulation in leaves under control and salt stress conditions. **D** Plant height measurements showing growth inhibition under salinity. **E** Principal component analysis (PCA) showing separation between control and salt-treated samples. CH: Total chlorophyll content, PH: plant height, RWC: Relative water content, FW: fresh weight, DW: dry weight, RDW: Root dry weight, RFW: Root fresh weight. **F** Correlation matrix of measured physiological parameters. **G** Salt tolerance indices were calculated across rice cultivars. **H** Heatmap visualization of log₂-transformed NaCl/CK ratios for key physiological parameters across cultivars. Data are presented as means ± SE (*n* = 3). Asterisks indicate significant differences between control and salt treatments: ** *p* < 0.01; *** *p* < 0.001

Salt stress significantly affected the morpho-physiological characteristics of all four rice cultivars, with statistically significant differential responses observed among genotypes (Fig. 1). Fresh weight measurements revealed substantial reductions across all cultivars in response to 200 mM NaCl treatment (*p* < 0.001, Fig. 1A). Under control conditions, fresh weights ranged from 0.16 to 0.49 g, with considerable variation among cultivars. However, salt stress exposure resulted in dramatic biomass reductions, with final fresh weights declining to 0.08–0.26 g depending on cultivar. Giza 178 exhibited the most pronounced decrease in fresh weight (48% reduction from control), declining from 0.16 g to 0.08 g under saline conditions. Conversely, Sakha 108 demonstrated superior biomass maintenance, retaining 0.26 g fresh weight under salt stress (a 45% reduction), which was significantly higher than that of all other cultivars tested.

Relative water content showed variable responses to salt treatment across tested cultivars (*p* < 0.001, Fig. 1B), reflecting cultivar-specific differences in osmotic stress management under high salinity. Under control conditions, all cultivars maintained RWC values between 81 and 94%, indicating optimal tissue hydration. Salt stress induced differential changes in water status among cultivars, with RWC values ranging from 67 to 88%. Notably, Sakha 108 exhibited water retention, maintaining an RWC of 84.5% under salt stress, representing a moderate decrease relative to control conditions. Giza 177 demonstrated a remarkable water-retention capacity, with an RWC of 87.8% under salt stress, higher than control values. Sakha 104 showed an RWC of 73.4% under saline conditions. In contrast, Giza 178 exhibited the lowest RWC of 67.3% under salt stress, indicating the most significant challenge in maintaining water homeostasis among the tested cultivars.

Saline conditions induced marked proline accumulation across the cultivar panel (Fig. 1C), confirming proline’s role as a universal marker of the osmotic stress response in rice. Under control conditions, basal proline levels remained low across all cultivars (2.7–3.8 µmol g⁻¹ FW). Salt stress triggered dramatic increases in proline biosynthesis, with accumulation levels ranging from 16.8 to 24.5 µmol g⁻¹ FW across cultivars. Giza 178 exhibited the strongest osmoprotective response with an 8.0-fold increase in proline content (from 3.1 to 24.5 µmol g⁻¹ FW), followed closely by Sakha 104 with a 6.6-fold elevation (from 3.7 to 24.4 µmol g⁻¹ FW). These pronounced increases suggest that Giza 178 and Sakha 104 rely heavily on proline-mediated osmotic adjustment as their primary strategy for mitigating stress. In contrast, Sakha 108 showed a more moderate 6.2-fold increase in proline accumulation (from 2.7 to 16.8 µmol g⁻¹ FW), suggesting that this cultivar may employ alternative or complementary stress tolerance mechanisms that reduce its dependence on extensive proline synthesis. Giza 177 exhibited a 5.6-fold increase in proline accumulation (from 3.4 to 17.4 µmol g⁻¹ FW), representing the lowest fold-change among the tested cultivars.

Salt stress resulted in significant growth inhibition across all cultivars, as evidenced by reduced plant height (Fig. 1D). Statistical analysis revealed highly significant treatment effects for all genotypes (*p* < 0.01 for Giza 178 and Sakha 104; *p* < 0.001 for Giza 177 and Sakha 108). Under control conditions, plant heights ranged from 32 to 66 cm, reflecting inherent genetic variation in growth vigor among the tested cultivars. Salt stress exposure caused substantial growth suppression, with plant heights declining to 21–57 cm. Giza 178 experienced the most severe growth inhibition, with plant height decreasing from 32 cm to 21 cm (34% reduction). Giza 177 also showed a pronounced reduction in height from 66 cm to 57 cm (14% reduction). The Sakha cultivars demonstrated greater resilience to salt-induced growth inhibition, with Sakha 108 maintaining 42 cm height under stress (compared to 50 cm under control) (16% reduction) and Sakha 104 retaining 41 cm height (compared to 62 cm under control) (34% reduction). These differential growth responses indicate that Giza cultivars are more sensitive to salinity-induced developmental constraints than Sakha cultivars. However, Sakha 104 showed a percentage height reduction similar to that of Giza 178.

Principal component analysis was performed to visualize the overall physiological response patterns across cultivars and treatments (Fig. 1E). PCA revealed clear separation between control (CK) and salt-treated (NaCl) samples along the first principal component (PC1), which explained 75.2% of the total variation in the multivariate physiological dataset comprising fresh weight, RFW, dry weight, RDW, relative water content, plant height, proline content, and chlorophyll content. This pronounced separation along PC1 highlights the dominant effect of salt stress on the overall physiological status of rice seedlings, overwhelming cultivar-specific baseline differences. The loading vectors indicate that growth-related parameters (fresh weight, RFW, dry weight, RDW, plant height, RWC, and chlorophyll) contribute positively to PC1 separation, while proline content loads in the opposite direction, consistent with its role as a stress-responsive rather than growth-promoting metabolite. The second principal component (PC2) accounted for 8.8% of variance and captured cultivar-specific response patterns within treatment groups. Control samples clustered tightly in the positive PC1 region, indicating physiological uniformity under optimal growth conditions. In contrast, salt-treated samples dispersed along both PC1 and PC2, reflecting both the universal stress response and cultivar-specific adaptive strategies. Notably, Sakha 108 salt-treated samples were positioned closer to the control cluster than those of other cultivars, suggesting that this genotype maintains physiological homeostasis more effectively under saline conditions.

Correlation analysis of measured physiological parameters revealed coordinated stress-response networks (Fig. 1F). The correlation matrix showed strong positive relationships among growth-related parameters, with fresh weight significantly correlated with dry weight (*r* = 0.89), relative water content (*r* = 0.70), plant height (*r* = 0.82), and chlorophyll content (*r* = 0.84). The RFW exhibited strong positive correlations with shoot biomass parameters and RWC (*r* = 0.60), indicating coordinated whole-plant responses to salt stress. RDW showed similar correlation patterns, confirming that root system maintenance is integral to overall salt tolerance. These positive correlations suggest that photosynthetic capacity, water status, and biomass accumulation are functionally integrated components of the growth response. Chlorophyll content exhibited moderate to strong positive correlations with other growth parameters, including RWC (*r* = 0.72), plant height (*r* = 0.66), and dry weight (*r* = 0.79), confirming that maintenance of photosynthetic pigments contributes to overall stress tolerance. Relative water content showed positive associations with all growth-related metrics, indicating that water retention capacity constitutes a critical determinant of physiological stability under saline conditions. In contrast, proline content showed consistent negative correlations with all growth and photosynthesis-related parameters, including fresh weight (*r* = -0.71), dry weight (*r* = -0.64), RFW (*r* = -0.58), RDW (*r* = -0.55), chlorophyll content (*r* = -0.45), RWC (*r* = -0.58), and plant height (*r* = -0.53). These inverse relationships reflect metabolic trade-offs associated with stress adaptation, in which resources are redirected from growth processes and photosynthetic machinery toward the synthesis of Osmo protectant defenses. The negative correlation between proline accumulation intensity and growth parameters suggests that, while proline accumulation serves as an adaptive stress response, excessive reliance on this mechanism may impose energetic costs that limit growth potential and photosynthetic performance under stress.

Integrated salt tolerance indices were calculated based on multiple physiological parameters to provide a comprehensive assessment of cultivar performance under saline conditions (Fig. 1G). The tolerance index was calculated based on maintenance of fresh weight, RWC retention, and growth performance relative to control conditions. This multidimensional approach identified Sakha 108 as the most salt-tolerant cultivar with a tolerance index of 78.6%, reflecting its superior ability to maintain biomass, water status, and growth under 200 mM NaCl stress. Giza 178 ranked second with a tolerance index of 62.2%, demonstrating moderate salt tolerance despite pronounced proline accumulation. Giza 177 exhibited a tolerance index of 56.5%, indicating moderate sensitivity to salt stress. Sakha 104 showed the lowest tolerance index (44.5%) among the tested cultivars, indicating high sensitivity to salinity despite its documented drought tolerance, underscoring distinct physiological mechanisms underlying tolerance to different abiotic stresses. This ranking (Sakha 108 > Giza 178 > Giza 177 > Sakha 104) establishes a quantitative framework for interpreting subsequent metabolomic differences and provides validation of the cultivar classifications.

The heatmap analysis (Fig. 1H) provided a comprehensive visualization of the differential physiological responses across cultivars under salt stress, with log₂-transformed NaCl/CK ratios revealing the magnitude and direction of changes. Growth-related parameters displayed negative log₂ values, confirming significant reductions under salinity as documented in Figs. 1A-D. Fresh weight showed log₂ ratios ranging from − 1.16 to -0.76, with Sakha 104 experiencing the most severe biomass loss (log₂ = -1.16, representing a 55% reduction) and Giza 177 showing the least decline (log₂ = -0.76, 41% reduction). Plant height exhibited log₂ ratios from − 0.60 to -0.21, with Giza 178 and Sakha 104 showing the greatest growth suppression (log₂ = -0.59 and − 0.60, respectively), while Sakha 108 and Giza 177 demonstrated superior height maintenance (log₂ = -0.24 and − 0.21, respectively). Relative water content displayed predominantly negative log₂ ratios ( -0.26 to -0.15) in three cultivars (Giza 178, Sakha 104, Sakha 108), reflecting impaired water homeostasis (10–16% reductions), while Giza 177 uniquely exhibited a modest positive log₂ value (+ 0.13), representing enhanced water retention capacity (9% increase). In stark contrast, proline accumulation displayed strongly positive log₂ ratios (2.37–3.02), representing 5.2- to 8.1-fold increases, with Giza 178 exhibiting the most robust osmoprotective response (log₂ = 3.02, 8.1-fold increase), followed by Sakha 104 (log₂ = 2.71, 6.5-fold) and Sakha 108 (log₂ = 2.68, 6.4-fold). Among the tested cultivars, Sakha 108 emerged as the most salt-tolerant genotype (tolerance index: 78.6%), demonstrating superior maintenance of biomass, growth resilience, and efficient water retention under 200 mM NaCl stress. Although Giza 178 exhibited the strongest biochemical stress response with the highest proline accumulation (8.1-fold, log₂ = 3.02), this cultivar ranked second in overall tolerance (62.2%), suggesting that excessive investment in osmoprotective mechanisms may impose metabolic costs that compromise growth and biomass production.

### Metabolomic data quality assessment and model validation

**Fig. 2:**
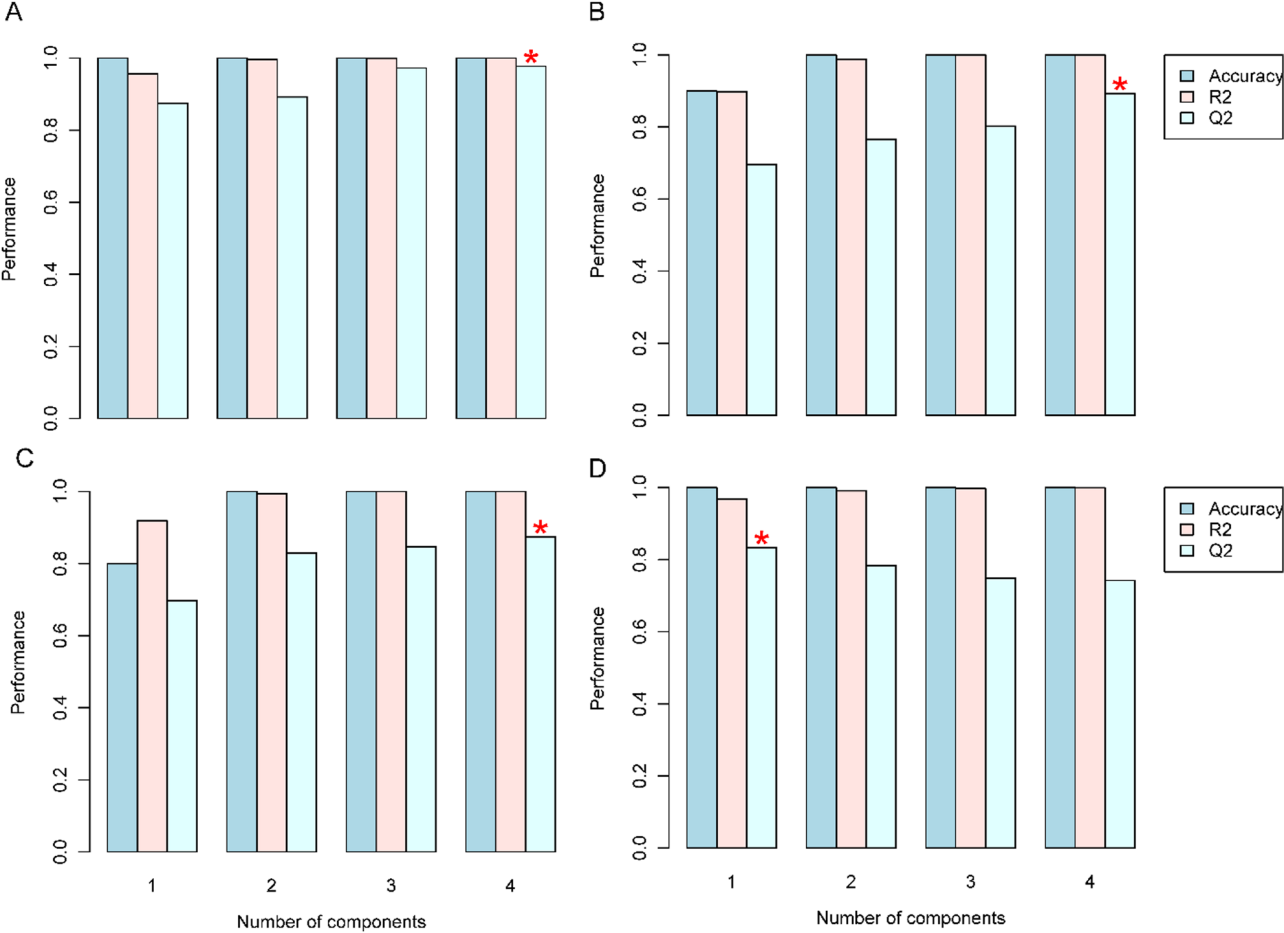
Orthogonal partial least squares discriminant analysis (OPLS-DA) model validation for metabolomic data. (**A**) Cross-validation and permutation test results for leaf tissue (**A**-Giza177, **B**- Giza178, **C**-Sakha104, **D**- Sakha108) showing R² and Q² values exceeding 0.7, confirming model validity and reliability

**Fig. 3:**
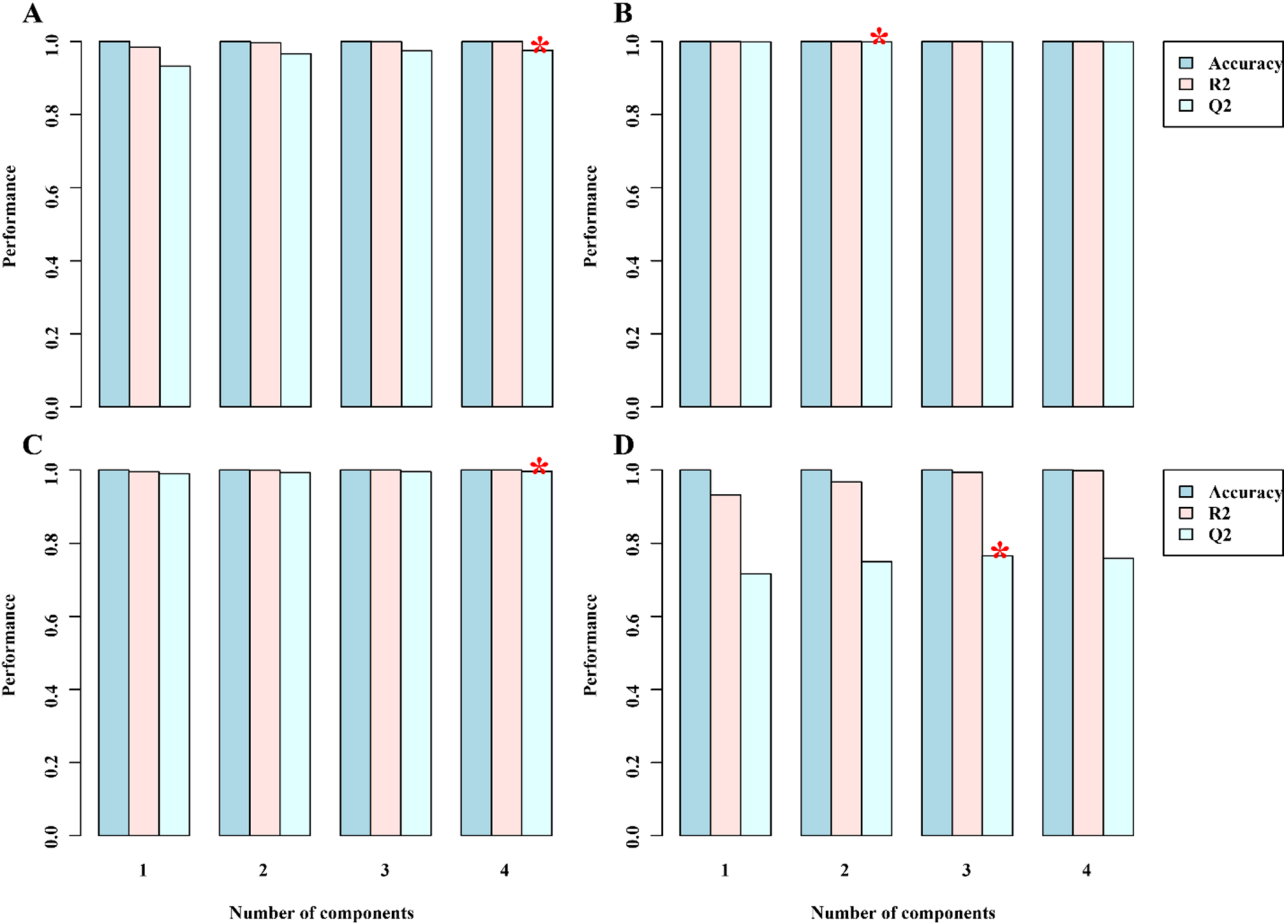
Cross-validation and permutation test results for root tissue (**A**-Giza177, **B**-Giza178, **C**-Sakha104, **D**-Sakha108) showing R² and Q² values exceeding 0.7, confirming model validity and reliability. Quality control samples showed R² values > 0.97, indicating high data quality for metabolomic profiling of four rice cultivars under salt stress

The reliability of the metabolomic dataset was assessed using comprehensive quality control metrics and validation of multivariate models. The performance of the orthogonal partial least squares discriminant analysis (OPLS-DA) model was evaluated using cross-validation and permutation testing, with a focus on key parameters such as accuracy, R², and Q². Quality control assessment indicated high data reliability across all samples. The correlation coefficients (R²) for quality control samples consistently exceeded 0.97, approaching the ideal value of 1.0, which signifies high-quality raw data acquisition. The robust performance observed in leaf (Fig. 2A-D) and root (Fig. 3A-D) tissue samples confirms the reliability of the metabolomic profiling methodology. Multivariate analysis demonstrated distinct separation among treatment groups, with samples clustering according to their specific experimental conditions. The four rice cultivars (Giza 177, Giza 178, Sakha 104, and Sakha 108) exhibited distinct metabolomic profiles in response to salt stress, with biological replicates clustering closely within their respective treatment groups. The observed clustering patterns demonstrate significant experimental reproducibility and confirm the efficacy of salt stress treatments in inducing cultivar-specific metabolomic responses.

### Multivariate analysis of GC-MS metabolomic data reveals tissue-specific and cultivar-dependent responses

**Fig. 4:**
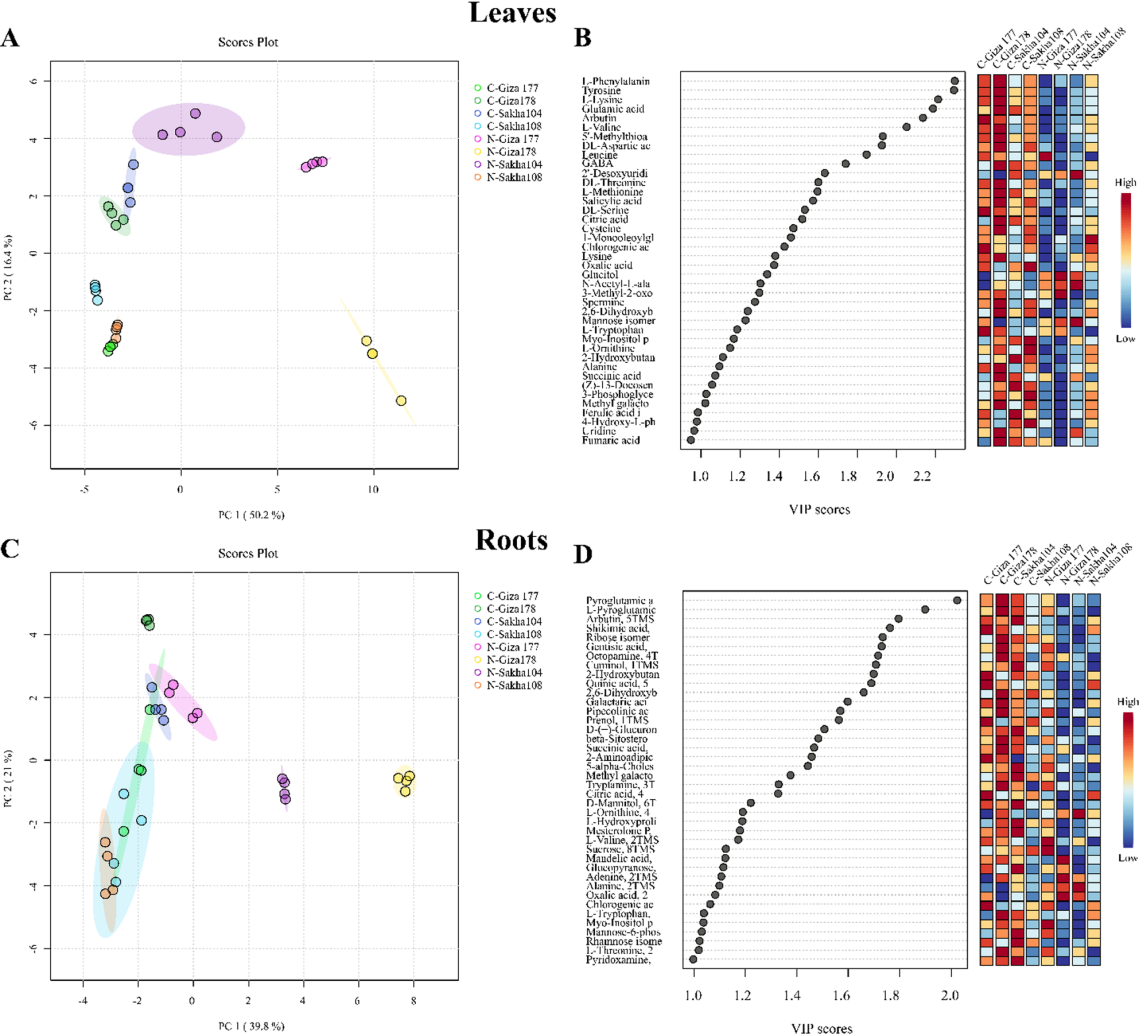
Principal Component Analysis (PCA) and Variable Importance in Projection (VIP) analysis of metabolomic profiles from four rice cultivars under control and salt stress conditions based on GC-MS data. **A** PCA score plot of leaf tissue metabolites with PC1 explaining 50.2% and PC2 accounting for 16.4% of total variance. **B** VIP score plot for leaf samples, illustrating metabolite importance rankings based on weighted sums of squares of PLS loadings and explained variance contributions. **C** PCA score plot of root tissue metabolites with PC1 explaining 39.8% and PC2 accounting for 21.0% of total variance. **D** VIP score plot for root samples, displaying the relative importance of individual metabolites in distinguishing treatment groups and cultivar responses

Principal component analysis was performed on GC-MS metabolomic data to assess variations in metabolite profiles among rice cultivars under control and salt stress conditions. PCA score plots were generated separately for leaf (Fig. 4A) and root (Fig. 4C) tissue samples to illustrate clustering patterns and treatment-induced metabolomic changes.

#### Leaf tissue metabolomic responses

Analysis of leaf tissue metabolomics revealed moderate distinction between salt-treated and control samples, with the first two principal components accounting for 66.6% of the total variance (PC1: 50.2%, PC2: 16.4%). Clustering patterns identified an overlap between Sakha104 and Giza178 under control conditions, while convergent metabolomic profiles were observed between salt-stressed Sakha108 and control Giza177 samples. These overlapping patterns indicate common baseline metabolomic characteristics among specific cultivars and the potential for stress-induced metabolomic convergence.

#### Root tissue metabolomic responses

Root tissue analysis revealed clearer separation patterns, with the first two principal components accounting for 60.8% of the total variance (PC1: 39.8%, PC2: 21.0%). While there was general overlap among the treatment groups, distinct metabolomic differentiation was evident for Sakha104 and Giza178 under salt-stress conditions and for Giza178 under control conditions. This tissue-specific response pattern suggests that root metabolomic profiles may be more sensitive to cultivar-specific salt-stress adaptations than leaf tissues. The PCA models demonstrated sufficient explanatory power, with each tissue type accounting for over 60% of the metabolomic variance, thereby validating the effectiveness of the multivariate analysis approach. Statistical significance of metabolite loadings was validated using Kruskal-Wallis ANOVA with false discovery rate (FDR) correction at α = 0.05.

#### Variable Importance in Projection (VIP) analysis

VIP scores serve as quantitative measures of metabolite significance within the analytical framework. Values exceeding 1.0 indicate substantial importance, while scores below 1.0 suggest reduced relevance to the experimental design. The metabolomic analysis identified 40 metabolites with VIP scores > 1.0 in both leaf tissues (Fig. 4B) and root systems (Fig. 4D) via GC-MS profiling. These metabolites significantly contributed to the phenotypic variations observed among cultivars under salinity stress. A VIP threshold of > 1.6 was applied to identify metabolites with optimal discriminatory capacity, resulting in the selection of 13 significant metabolites that contributed to notable inter-group variance. This subset represents the primary biochemical factors underlying the differential stress responses observed among the four cultivars. The complete metabolomic dataset comprised 114 polar metabolites from leaf tissues and 97 from root systems (Tables S1 and S2), demonstrating the substantial metabolic complexity captured through GC-MS analysis.

### Cultivar-specific metabolic responses to salt stress

Cultivar-specific metabolic responses to salinity stress revealed unique biochemical signatures across the tested genotypes. In Giza 177, elevated leucine and succinic acid concentrations were observed under saline conditions compared to control treatments and other cultivars, suggesting enhanced amino acid metabolism and tricarboxylic acid cycle activity. Conversely, Sakha108 demonstrated preferential accumulation of 1-monooleoglycerol compounds and chlorogenic acid in leaf tissues under salt stress, indicating activation of lipid metabolism and phenolic compound biosynthesis pathways. Root tissue analysis revealed distinct cultivar-specific responses, with Giza177 exhibiting significantly higher concentrations of L-valine and L-pyroglutamic acid under saline conditions compared to controls and other cultivars. Additionally, increased citric acid and arbutin accumulation were observed across cultivars under salt stress, indicating shared metabolic adjustments related to organic acid metabolism and phenolic glycoside production in response to osmotic and ionic stress.

#### Hierarchical clustering and comparative analysis

**Fig. 5:**
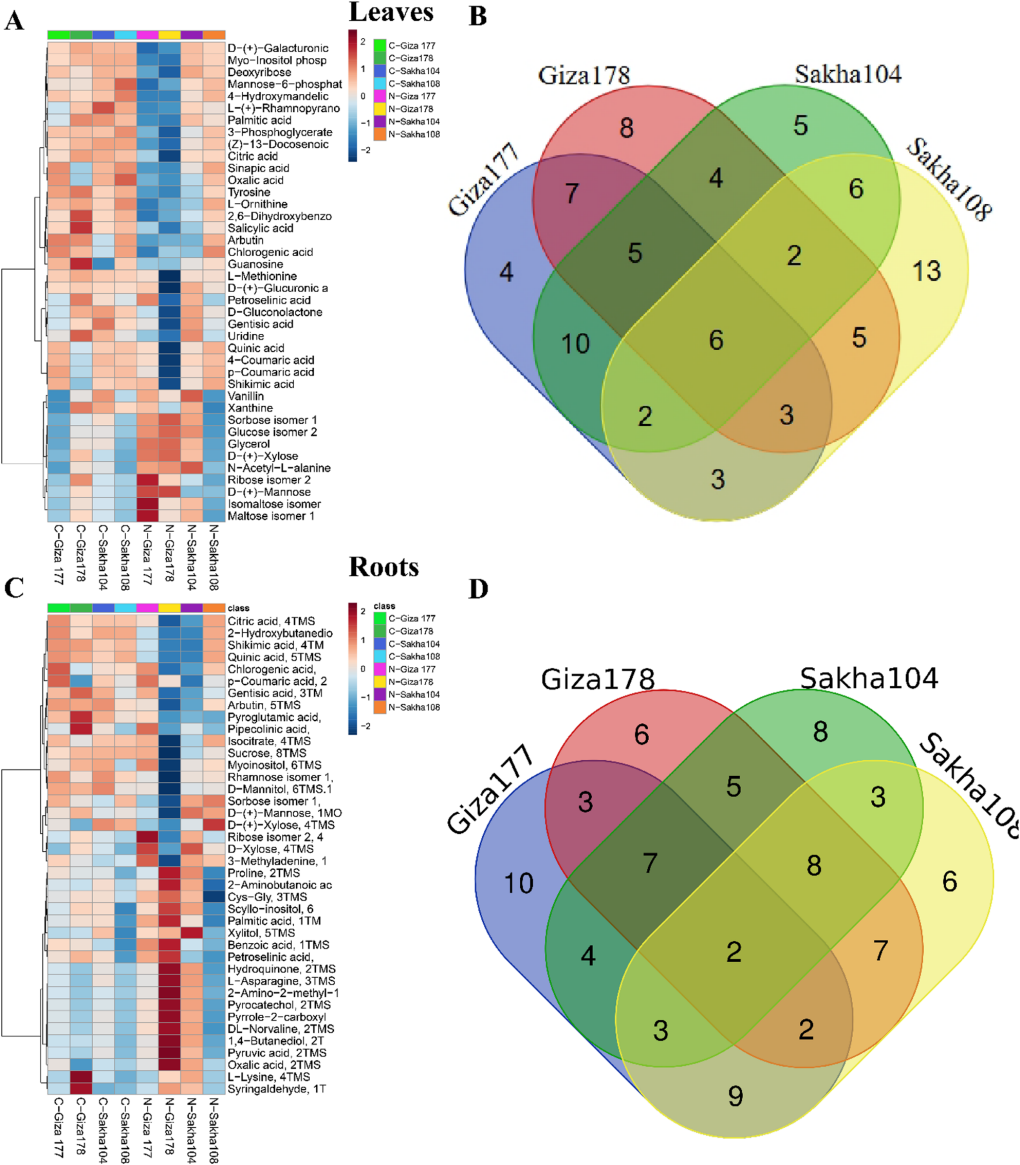
Metabolomic profiling and comparative analysis of salinity stress responses across four rice cultivars. Heatmaps display hierarchical clustering of the top 40 most variable metabolites based on VIP scores, with experimental conditions arranged in rows and metabolite profiles in columns. Color intensity represents relative metabolite concentrations, with red indicating elevated abundance and blue reduced levels. **A** Leaf tissue metabolomic heatmap analysis. **B** Venn diagram illustrating shared and unique metabolites among cultivars at the leaf level. **C** Root tissue metabolomic heatmap analysis. **D** Venn diagram showing metabolite distribution patterns among cultivars at the root level

Hierarchical clustering analysis of metabolomic profiles revealed clear tissue-specific responses to salinity stress at both foliar and root levels. Heatmap visualization utilized a matrix layout with rows representing experimental conditions (control versus salt-treated samples) and columns displaying individual metabolite profiles. Metabolite concentrations were represented using a color-coded intensity scale, where red indicates high abundance and blue indicates lower concentrations relative to the dataset’s meaning. A detailed heatmap analysis of the top 40 VIP-selected metabolites provided a clear visualization of concentration dynamics across the various treatment conditions. Leaf-level responses are depicted in Fig. 5A, while root-level patterns are presented in Fig. 5C. This analysis identified tissue-specific and cultivar-dependent metabolic reprogramming in response to salinity stress, highlighting the complex mechanisms underlying plant stress adaptation. Venn diagram analysis was employed to identify both shared and unique metabolomic signatures across the four rice cultivars, enabling characterization of cultivar-specific stress responses. Among the 114 metabolites identified in leaf tissues, six metabolites demonstrated consistent expression patterns across all four cultivars (Fig. 5B), indicating conserved metabolic pathways associated with foliar salinity tolerance. Root metabolomic analysis of 97 identified compounds revealed that only two metabolites exhibited universal expression patterns across cultivars (Fig. 5D), suggesting higher metabolic diversity in root tissue responses to osmotic stress.

#### Hierarchical clustering analysis of rice cultivar metabolomic profiles

**Fig. 6:**
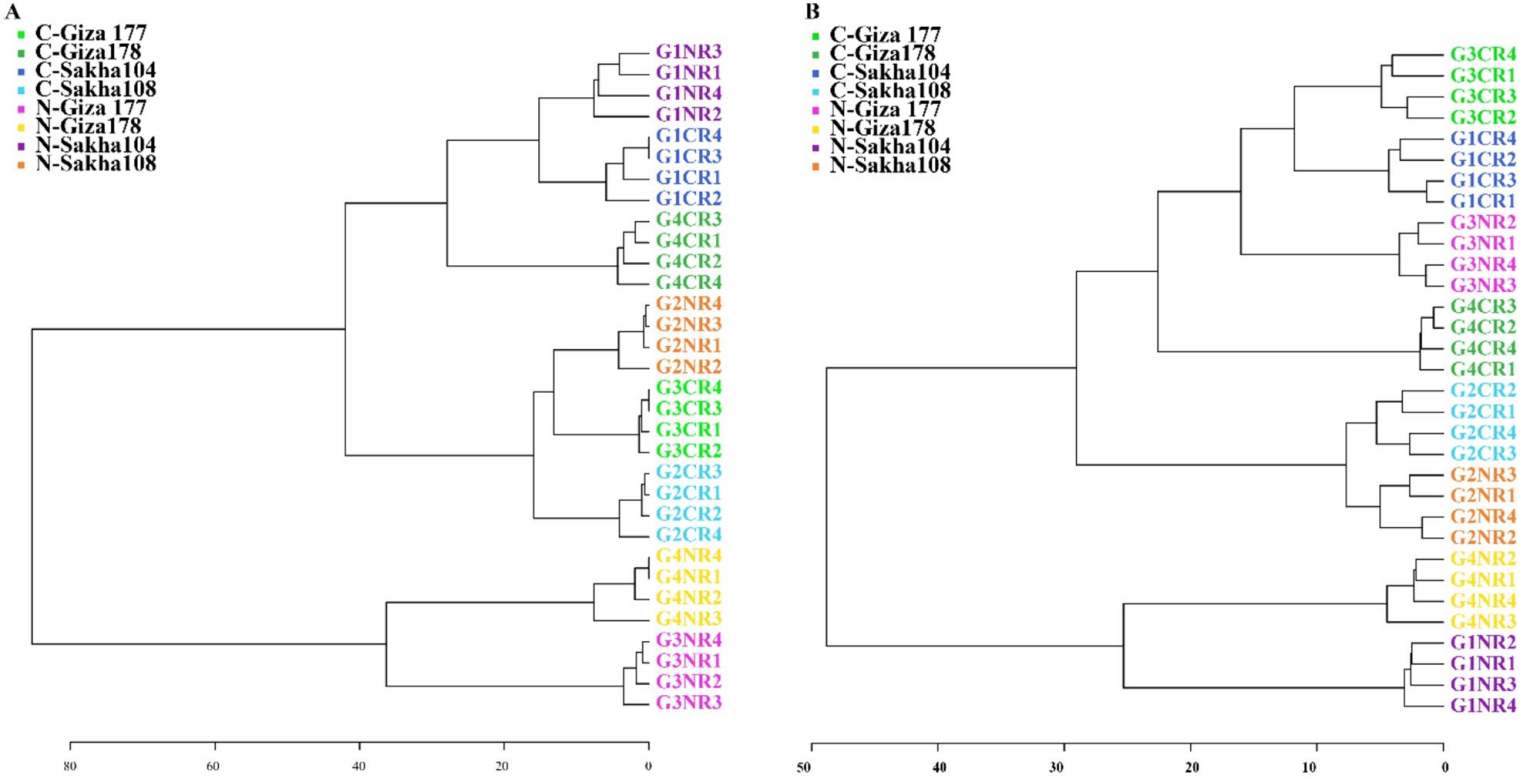
Hierarchical cluster analysis of metabolomic profiles in rice cultivars under salt stress conditions. Dendrograms showing hierarchical clustering of metabolomic profiles from (**A**) leaf tissues and (**B**) root tissues of four rice cultivars (Giza 177, Giza 178, Sakha 104, Sakha 108) under control (C-) and salt stress (N-) conditions. Color coding represents different cultivars: green (Giza 177), blue (Giza 178), pink (Sakha 104), and orange (Sakha 108). Numbers following cultivar names indicate biological replicates (1–4). Clustering was performed using Euclidean distance and Ward’s linkage method based on normalized metabolite intensities

Hierarchical clustering of metabolomic profiles revealed distinct tissue-specific patterns and clear treatment-induced metabolic reorganization across the four rice cultivars under salt stress. In leaf tissues (Fig. 6A), the dendrogram showed a clear separation between control and salt-treated samples, with metabolomic profiles clustering primarily by treatment status rather than cultivar identity. This clustering pattern supports the finding that foliar metabolism exhibits more conserved stress responses, with samples forming two major clusters corresponding to control (C-) and salt-treated (N-) conditions. Within each treatment group, cultivar-specific sub-clusters were observed, indicating that while the overall stress response is conserved in leaves, subtle cultivar-specific metabolic adjustments still occur.

The root tissue clustering analysis (Fig. 6B) revealed a more complex metabolic landscape, with greater inter-cultivar variation and less distinct treatment-based separation than in leaf tissues. The dendrogram indicates that root metabolism shows greater plasticity and cultivar-specific adaptation strategies under salt stress. Unlike the clear treatment-based clustering observed in leaves, root samples showed mixed clustering patterns, in which cultivar identity played a more prominent role in determining metabolic similarity. This observation aligns with the finding that root tissues exhibited 2-fold greater metabolic diversity than leaves, supporting their role as the primary site for stress perception and adaptive metabolic responses.

Notably, Sakha 108 samples (represented in orange) frequently formed distinct clusters separate from those of other cultivars in both tissues, reflecting its unique metabolic signature and superior salt tolerance (78.6% tolerance index). The clustering patterns of Giza 177 (green) and Giza 178 (blue) showed intermediate positioning between the highly tolerant Sakha 108 and the more sensitive Sakha 104 (pink), which correlates with their moderate tolerance indices of 62.2% and 56.5%, respectively. These hierarchical relationships provide visual confirmation of the cultivar performance ranking and demonstrate that metabolomic profiles can effectively discriminate between salt tolerance levels, supporting the utility of the identified metabolic biomarkers for breeding applications and cultivar selection programs.

#### Volcano plot analysis of differential metabolite expression under salt stress

**Fig. 7:**
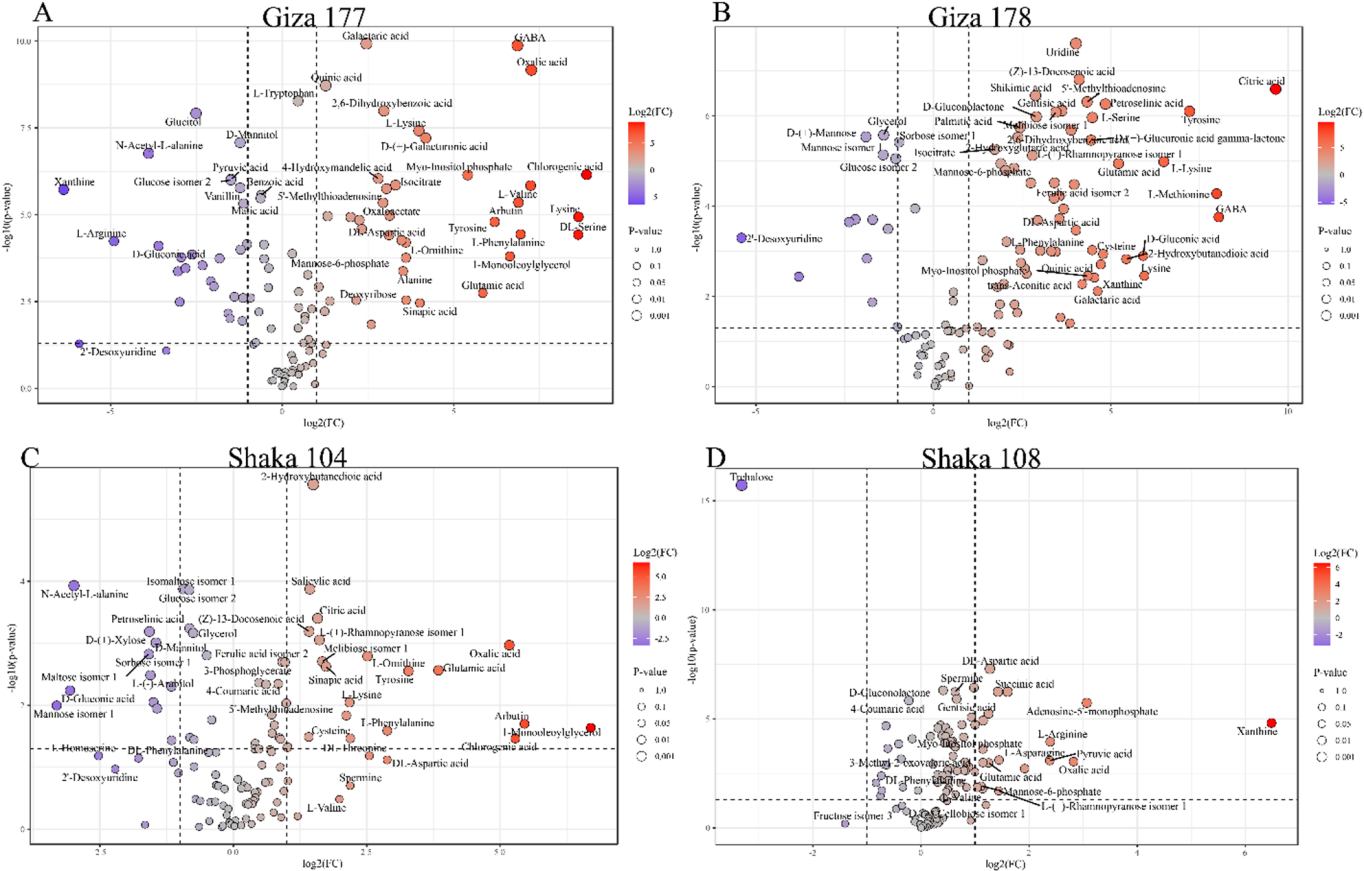
Differential metabolite accumulation in leaf tissues of rice cultivars under salt stress. Volcano plots showing log2(fold change) vs. -log10(p-value) for metabolites in leaf tissues of (**A**) Giza 177, (**B**) Giza 178, (**C**) Sakha 104, and (**D**) Sakha 108 under 200 mM NaCl treatment compared to control conditions. Red points indicate significantly upregulated metabolites, blue points indicate significantly downregulated metabolites, and gray points represent non-significant changes. Vertical dashed lines represent fold-change thresholds (± 1.5-fold), and horizontal dashed lines represent statistical significance thresholds (*p* < 0.05). Point sizes reflect p-values, with larger points indicating higher statistical significance

Volcano plot analysis revealed distinct cultivar-specific metabolic responses to salt stress, with each rice variety demonstrating unique patterns of metabolite regulation. Giza 177 (Fig. 7A) exhibited the most pronounced metabolic perturbation under salt treatment, with numerous significantly upregulated metabolites, including GABA, oxalic acid, galactaric acid, and quinic acid, showing high fold-changes (log2FC > 2) and statistical significance (-log10(p-value) > 2). Notable upregulated compounds included amino acids such as L-tryptophan and L-lysine, organic acids like 2,6-dihydroxybenzoic acid, and sugar metabolites, indicating broad activation of stress-responsive pathways. Conversely, several metabolites, including xanthine, glucose isomers, and N-acetyl-L-alanine, were significantly downregulated, suggesting metabolic reallocation under stress conditions.

Giza 178 (Fig. 7B) demonstrated a more moderate but still substantial metabolic response, with citric acid, uridine, and various organic acids showing significant upregulation. The cultivar displayed enhanced production of amino acids, including L-serine, tyrosine, and L-methionine, as well as increased accumulation of sugar derivatives and organic acids, such as (Z)-13-docosenoic acid and shikimic acid. Interestingly, GABA and several phosphate-containing compounds were among the significantly upregulated metabolites, while 2’-deoxyuridine and specific glucose isomers were downregulated, indicating selective activation of a metabolic pathway.

Sakha 104 (Fig. 7C) exhibited a distinct metabolic profile, characterized by moderate fold changes but significant regulation of key metabolites. The cultivar exhibited upregulation of organic acids, including citric acid, salicylic acid, and oxalic acid, as well as amino acids such as L-valine and tyrosine. Notably, 2-hydroxybutanedioic acid emerged as a highly significant upregulated metabolite, while compounds like 2-deoxyuridine, N-acetyl-L-alanine, and various glucose isomers were downregulated. The overall metabolic response pattern suggested a more controlled adaptation than in the Giza varieties.

Sakha 108 (Fig. 7D), the most salt-tolerant cultivar, exhibited a remarkably restrained metabolic response, with fewer significantly altered metabolites and generally lower fold changes compared to other cultivars. Trehalose emerged as the most dramatically upregulated metabolite, indicating activation of osmotic adjustment mechanisms. Different compounds were significantly upregulated, including succinic acid, adenosine-5-monophosphate, and several organic acids, while xanthine was downregulated. The relatively modest metabolic perturbation in Sakha 108, combined with the strategic upregulation of key stress-protective metabolites, such as trehalose, suggests a more efficient, targeted stress response mechanism that contributes to its superior salt tolerance. This pattern contrasts sharply with the extensive metabolic disruption observed in less tolerant varieties, supporting the hypothesis that effective salt tolerance involves precise metabolic regulation rather than broad-scale metabolic activation.

#### Root tissue metabolomic response to salt stress: volcano plot analysis

**Fig. 8:**
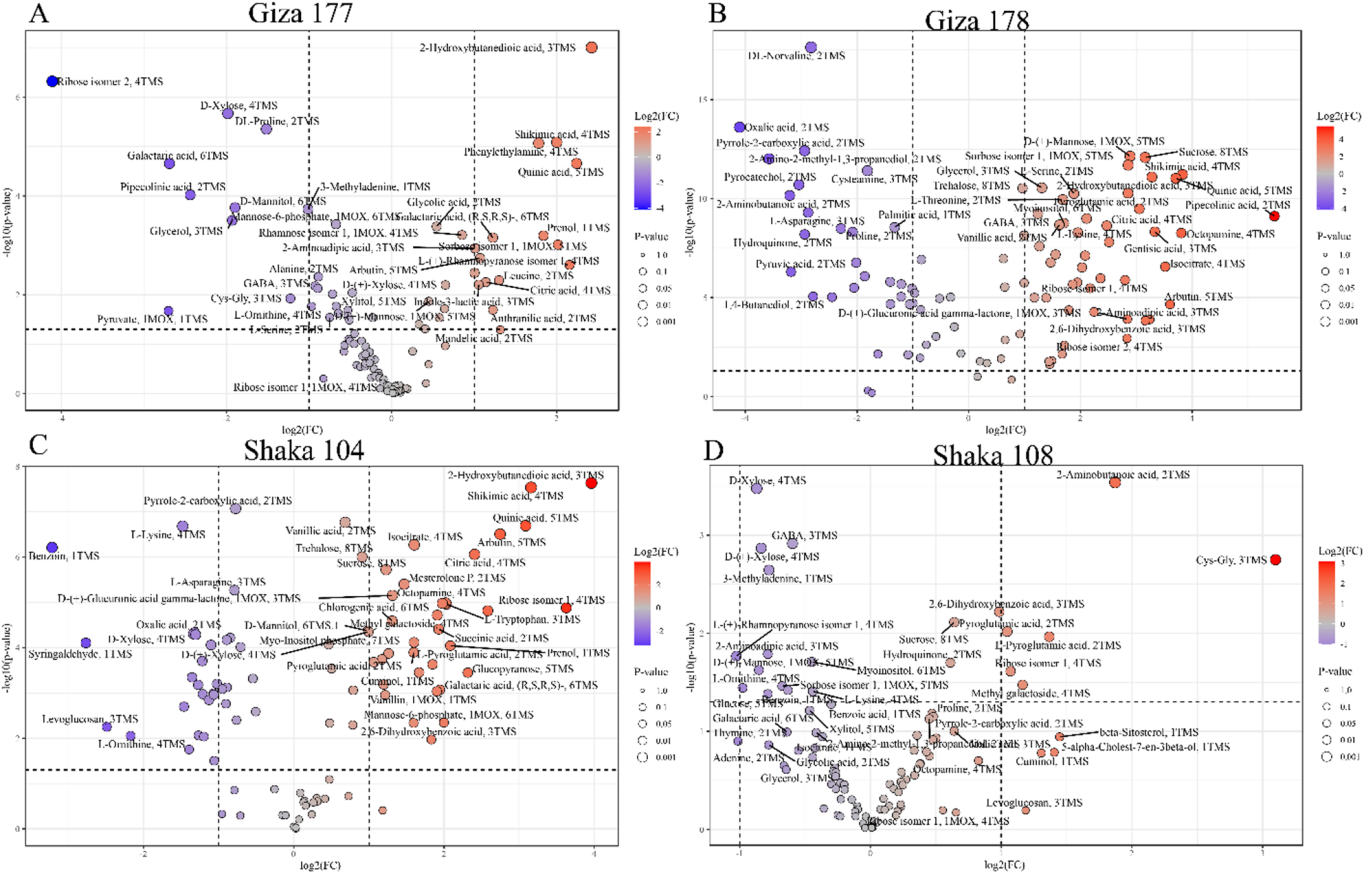
Root tissue metabolomic responses to salt stress across rice cultivars. Volcano plots displaying differential metabolite accumulation in root tissues of (**A**) Giza 177, (**B**) Giza 178, (**C**) Sakha 104, and (**D**) Sakha 108 following 14-day exposure to 200 mM NaCl. The X-axis shows log2(fold change) relative to control conditions, and the Y-axis shows -log10(p-value) for statistical significance. Color gradient represents fold-change magnitude: red indicates upregulation, blue indicates downregulation. Metabolite names are labeled for compounds exceeding significance thresholds (|log2FC| > 1, *p* < 0.05). Point sizes correspond to p-value significance levels

Volcano-plot analysis of root tissue metabolomes revealed profound cultivar-specific metabolic reprogramming under salt stress, with each variety demonstrating distinct adaptation strategies at the metabolic level. Giza 177 (Fig. 8A) exhibited extensive metabolic perturbation with numerous highly significant upregulated metabolites, including 2-hydroxybutanedioic acid as the most dramatically increased compound (log2FC > 4), alongside shikimic acid, quinic acid, and phenylethylamine showing substantial fold-changes. The cultivar demonstrated strong activation of amino acid metabolism pathways, as evidenced by the significant upregulation of glycolic acid, citric acid, and various organic acids. In contrast, ribose isomers and several sugar metabolites were notably downregulated. This broad metabolic response pattern suggests an energy-intensive adaptation strategy involving multiple biochemical pathways.

Giza 178 (Fig. 8B) exhibited a more focused yet substantial metabolic response, with DL-norvaline, oxalic acid, and sorbose isomers among the most significantly upregulated metabolites. The cultivar exhibited enhanced production of amino acids, including L-serine and L-threonine, as well as various organic acids, such as pyruvate-2-carboxylic acid and quinic acid. Notably, GABA and octopamine emerged as key upregulated stress-responsive compounds, while several ribose isomers and sugar derivatives were downregulated. The metabolic profile indicates that amino acid biosynthesis and organic acid metabolism are primary adaptation mechanisms.

Sakha 104 (Fig. 8C) demonstrated a moderate metabolic adjustment, with 2-hydroxybutanedioic acid and shikimic acid as the prominently upregulated metabolites. The cultivar exhibited increased production of trehalose, citric acid, and various amino acids, including L-tryptophan and L-asparagine, suggesting activation of osmotic adjustment and protein metabolism pathways. Several organic acids and phenolic compounds were significantly upregulated, while levoglucosan and specific sugar isomers were downregulated. The metabolic response pattern indicates a balanced approach to stress adaptation with emphasis on osmoprotectant accumulation.

Sakha 108 (Fig. 8D), the most salt-tolerant cultivar, exhibited a remarkably controlled metabolic response, characterized by fewer metabolites with dramatic changes and more moderate fold changes than in other varieties. D-xylose and 2-aminobutanoic acid emerged as the most significantly upregulated compounds, while GABA, Cys-Gly, and 3-methyladenine showed notable increases. The cultivar demonstrated strategic upregulation of specific amino acids and organic acids, including L-pyroglutamic acid and hydroquinone, while maintaining relatively stable levels of most other metabolites. This restrained yet targeted metabolic adjustment, particularly the moderate upregulation of key osmoprotectants and signaling molecules, reflects an efficient stress response mechanism that likely contributes to its superior salt tolerance. The contrast between Sakha 108 controlled response and the extensive metabolic disruption observed in less tolerant varieties supports the concept that effective salt tolerance involves precise metabolic fine-tuning rather than wholesale metabolic reorganization, highlighting the importance of metabolic efficiency in stress adaptation.

## Discussion

### Differential physiological responses to salt stress among rice cultivars

The present study demonstrates significant inter-cultivar variation in salt tolerance mechanisms among the four tested rice cultivars, with Sakha108 exhibiting superior performance under saline conditions. Our findings align with previous reports indicating that salt tolerance in rice is a complex, multigenic trait with substantial genotypic variation [34–36]. The observed 78.6% salt tolerance index for Sakha108 is comparable to values reported for other salt-tolerant rice varieties such as Pokkali and CSR27, which typically maintain 70–80% of their growth parameters under moderate salinity stress [37, 38]. Within our experimental conditions, the significant reduction in fresh weight and RWC observed across all cultivars under salt stress is consistent with numerous studies demonstrating that osmotic stress and ion toxicity are primary mechanisms limiting plant growth under saline conditions [39–41]. However, the differential response patterns observed among the tested cultivars suggest distinct adaptive strategies. Sakha104 and Sakha108 exhibited significantly higher RWC values than the Giza cultivars, suggesting potentially superior osmotic adjustment capacity in this study. This finding corroborates previous research by Kamanga [41] who reported that salt-tolerant rice varieties typically maintain higher tissue water content through enhanced K+/Na + selectivity and compartmentalization mechanisms.

### Metabolomic insights into salt tolerance mechanisms

GC-MS metabolomic analysis revealed distinct tissue-specific responses to salt stress, with root tissues showing greater metabolic diversity compared to leaves in the tested cultivars. Root tissues demonstrated higher cultivar-specific metabolic plasticity compared to leaves, evidenced by: lower metabolite conservation across cultivars (2.1% vs. 5.3%), more distributed variance in PCA analysis (PC1: 39.8% in roots vs. 50.2% in leaves), and mixed hierarchical clustering patterns that prioritize cultivar identity over treatment status in root tissues (Fig. 6B), contrasting with the clear treatment-based separation observed in leaves (Fig. 6A). These quantitative metrics support the interpretation that root metabolism employs more diverse, cultivar-specific adaptation strategies. The identification of 114 metabolites versus 97 in roots, combined with different clustering patterns in PCA analysis, suggests that metabolic reprogramming occurs differentially across plant tissues within this experimental framework. This tissue-specific response pattern aligns with findings from Xu et al. [42] who demonstrated that root and shoot tissues employ distinct metabolic strategies during adaptation to abiotic stress. Based on our analysis of these four cultivars, the greater metabolic diversity seen in root responses, with only two metabolites shared across cultivars, contrasts with leaves, which have six conserved metabolites. This indicates that, among the tested genotypes, root metabolism may be more adaptable and rely on cultivar-specific mechanisms. This finding is consistent with the root system’s role as the primary site of salt perception and initial stress response [43, 44]. Similar tissue-specific metabolic differentiation has been reported in other stress studies, in which roots showed greater metabolic flexibility in response to drought and salinity [45].

The metabolomic analysis revealed distinct biochemical signatures for each tested cultivar, providing insights into their respective tolerance mechanisms. Our results suggest that Giza177’s accumulation of leucine and succinic acid under salt stress may indicate enhanced branched-chain amino acid metabolism and tricarboxylic acid (TCA) cycle activity. This response pattern is similar to that reported in salt-sensitive wheat varieties, where increased amino acid catabolism serves as an alternative energy source during stress [46, 47]. In contrast, our findings indicate that Sakha108’s preferential accumulation of 1-monooleoglycerol compounds and chlorogenic acid suggests potential activation of lipid metabolism and phenolic compound biosynthesis. The accumulation of phenolic compounds, particularly chlorogenic acid, has been associated with enhanced antioxidant capacity and cellular protection during salt stress [48]. This metabolic profile appears consistent with salt-tolerant varieties that maintain cellular integrity through enhanced antioxidant systems [49, 50]. The root-specific accumulation of L-valine and L-pyroglutamic acid in Giza177 indicates enhanced amino acid metabolism in below-ground tissues, potentially serving as nitrogen storage compounds during stress. Similar patterns have been observed in other cereals under salt stress, where amino acid accumulation in roots provides substrate for stress-responsive protein synthesis [51]. The identification of 13 metabolites with VIP scores greater than 1.6 provides a focused set of potential biomarkers for screening salt tolerance. This biomarker approach has been successfully applied in other crop species, where metabolite-based selection has accelerated breeding programs for stress tolerance [52]. The tissue-specific nature of these biomarkers suggests that both root and shoot sampling may be necessary for a comprehensive assessment of tolerance.

The findings of this study have direct implications for rice breeding and biotechnology applications. The identification of Sakha108 as a superior salt-tolerant variety provides valuable germplasm for breeding programs targeting saline-affected areas. The identified metabolomic biomarkers could accelerate screening processes, thereby reducing the time and resources required for traditional field-based evaluations. Furthermore, the mechanistic insights from this study could inform biotechnological approaches to enhance salt tolerance. The enhanced phenolic metabolism observed in tolerant varieties suggests that genetic engineering targeting phenylpropanoid pathway genes could improve salt tolerance [53, 54]. Similarly, differential amino acid metabolic patterns could guide metabolic engineering approaches to develop stress-resilient crops.

## Conclusions

Our combined morpho-physiological and metabolomic analysis shows that different rice cultivars use distinct molecular strategies to adapt to salt stress, which has implications for global food security. Sakha108 stands out as a promising salt-tolerant genotype due to an integrated stress response that balances osmoregulation, growth, and metabolic efficiency, reaching 78.6% stress tolerance without the energetic costs seen in other cultivars. Through detailed GC-MS metabolomics, we observed tissue-specific metabolic reprogramming, with roots exhibiting twice the metabolic diversity of leaves across genotypes, enhancing our understanding of plant stress adaptation hierarchies. The identification of 13 key metabolic biomarkers (VIP > 1.6) offers promising targets for breeding improvements. Our findings suggest that effective salinity tolerance results from coordinated multi-pathway networks, indicating complexity beyond single-gene effects. These initial results lay the groundwork for a precision phenotyping approach that could shift stress biology from descriptive to predictive, providing practical strategies for developing climate-resilient crops to feed growing populations on increasingly marginal lands. Nevertheless, validation across diverse genetic backgrounds and environments is necessary before applying these insights to breeding programs.

## Supplementary Information

Supplementary Material 1.

## Data Availability

All data generated or analyzed during this study are included in this published article. The datasets used and analyzed in the current study are available from the S.F.L. upon reasonable request.
